# Quantification and characterization of microplastics ingested by mangrove oysters across West Africa

**DOI:** 10.1007/s11356-024-34470-9

**Published:** 2024-08-01

**Authors:** Edem Mahu, Tyronne Jude Vanderpuye-Orgle, Charles Mario Boateng, Maurice Oti Edusei, Gabriella Akpah Yeboah, Ernest Obeng Chuku, Paulina Okpei, Isaac Okyere, David Dodoo-Arhin, Edward Akinnigbagbe Akintoye

**Affiliations:** 1 Department of Marine and Fisheries Sciences, University of Ghana, Accra, Ghana; 2 Institute for Marine and Antarctic Studies, University of Tasmania, Taroona, 7053 Australia; 3 University of Energy and Natural Resources, Sunyani, Ghana; 4 Department of Fisheries and Aquatic Sciences, School of Biological Sciences, CANS, University of Cape Coast (UCC), Cape Coast, Ghana; 5 Centre for Coastal Management, Africa Centre of Excellence in Coastal Resilience - (ACECoR), UCC, Cape Coast, Ghana; 6 Department of Material Science and Engineering, University of Ghana, Accra, Ghana; 7 Nigerian Institute for Oceanography and Marine Research, Lagos, VI Nigeria

**Keywords:** Mangrove oyster, Ecosystem, FTIR, Optical microscopy, Estuary, Lagoon

## Abstract

Microplastic ingestion by marine organisms presents a challenge to both ecosystem functioning and human health. We characterized microplastic abundance, shape, size, and polymer types ingested by the West African mangrove oyster, *Crassostrea tulipa* (Lamarck, 1819) sampled from estuaries and lagoons from the Gambia, Sierra Leone, Ghana, Benin, and Nigeria using optical microscopy and Fourier transform infrared (FTIR) techniques. A total of 780 microplastics were isolated in the whole tissues of the 250 oysters (*n* = 50 oysters per country). The abundance and distribution of microplastics in the oysters followed the pattern: the Gambia > Ghana > Sierra Leone > Nigeria > Benin. The Tanbi wetlands in the Gambia recorded the highest average of 10.50 ± 6.69 per oyster while the Ouidah lagoon in Benin recorded the lowest average of 1.80 ± 1.90 per oyster. Overall, microplastic numbers varied significantly (*p* < 0.05) among the five countries. Microfibers, particularly those within 1001–5000 μm size, dominated the total microplastic count with a few fragments and films. No spherical microplastics were isolated in the oysters. In the Sierra Leone and Benin oysters, fragments and films were absent in the samples. Microplastic between the 1001 and 5000 μm size class dominated the counts, followed by 501–1000 μm, 101–500 μm, and 51–100 μm. Five polymer groups namely polyethylene, polyester, nylon, polypropylene, and polyamide were identified across the five countries, with polyethylene occurring in oysters from all five countries and polyester occurring in all but the oysters from Nigeria. This diversity of polymers suggests varied sources of microplastics ingested by the studied oysters. The absence of microspheres across the five supports findings from other studies that they are the least ingested and highly egested by the oysters.

**Graphical Abstract:**

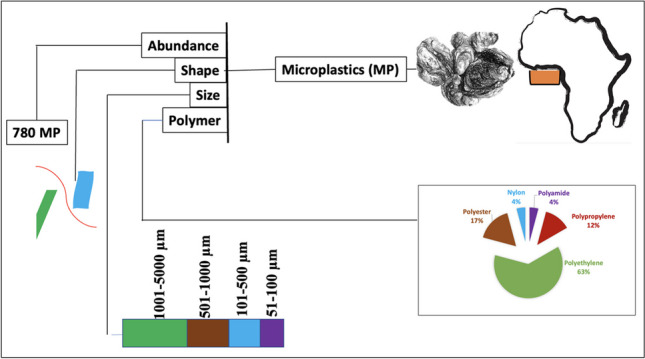

## Introduction

Microplastic pollution of the environment is a global problem that calls for urgent attention. In recent times, the far-reaching effects of global plastic production and utilization surge (Geyer et al. 2017), coupled with increased trends in the mismanagement of its waste (Jambeck et al. 2015), have led to a more complex and difficult problem of microplastic pollution of air, land, and water. Microplastic, defined as plastic with a length < 5 mm but > 1 μm (Anderson et al. 2016), can be grouped into primary (purposefully manufactured) or secondary (resulting from the breakdown of larger plastics) microplastics (Auta et al. 2017). They are ubiquitous and have been found in air (O’Brien et al. 2020; Syafei et al. 2019; Zhang et al. 2020), water (Egessa et al. 2020; La Daana et al. 2017; Luo et al. 2019; Lusher et al. 2014; Zhang et al. 2018), sediment (Chico-Ortiz et al. 2020; Gerolin et al. 2020; Van Cauwenberghe et al. 2013, 2015; Yao et al. 2019), soils (Rillig et al. 2017; Surendran et al. 2023; Wang et al. 2020; Yang et al. 2022a, b), plants (Bosker et al. 2019), animal tissues (Adika et al. 2020; Carlin et al. 2020; Feng et al. 2019; Hossain et al. 2019; Mahu et al. 2023; Pozo et al. 2019; Wang et al. 2021; Wootton et al. 2021), and human body parts (Huang et al. 2022; Ragusa et al. 2021; Schwabl et al. 2019; Wootton et al. 2021). Because of their microscopic and widespread nature, they are potentially bioavailable to a wide range of marine organisms and are easily ingested by low trophic suspension, filter and deposit feeders, detritivores, and planktivores (Browne et al. 2008; Graham & Thompson 2009; Koelmans et al. 2017; Murray & Cowie 2011). Accumulation of microplastics within marine organisms may result in physical harm, such as abrasions to internal organs and blockages (Wright et al. 2013). Leaching of additive chemicals and monomers from ingested microplastics could further result in carcinogenesis and endocrine disruption (Chenet et al. 2021; Manikkam et al. 2013; Oehlmann et al. 2009; Sax 2010). Microplastics have a higher affinity for hydrophobic contaminants including the persistent organic pollutants and other contaminants in the water, thus making them become a pathway of other contaminants into the food chain (Teuten et al. 2009).

Filter-feeding bivalves, including oysters, are among the most susceptible species to microplastic ingestion because of prey-size similarity (Germanov et al. 2018; Wright et al. 2013). Oysters filter large quantities of water through their gills to facilitate gas exchange and extract phytoplankton (Jørgensen 1996). This indispensable dependence on filter-feeding for respiration and feeding increases their exposure risk to contaminants in their habitats, thereby increasing their risk of ingesting microplastics and getting impacted by microplastics and their associated contaminants (Edge et al. 2014). Nonetheless, they can selectively ingest or egest different types of microplastics due to anatomical constraints of the gill, labial palps, and mouth. On the other hand, because of the filter-feeding and sedentary lifestyles of oysters, some studies have recommended them as excellent indicators of water contamination, including some types of microplastic in marine and estuarine systems (Zhu et al. 2020). Also, the wide geographical distribution of oysters makes them ideal specimens to monitor global levels of microplastics in the environment (Wootton et al. 2022).

Although an excellent food and livelihood source in several countries, oyster reefs have come under intense anthropogenic pressures including overharvesting, climate change, habitat loss, ocean acidification, toxin exposure, disease, pollution from heavy metals, persistent organic pollutants, sediments, and microplastics (Beck et al. 2011; Ducker & Falkenberg 2020; Kurochkin et al. 2009; Mahu et al. 2022; Van Cauwenberghe & Janssen 2014). Studies on microplastic consumption by oysters are rising owing to their vital ecological niche, economic importance, and potential to become exposure routes of contaminants to humans. In the wake of the growing consumption of bivalves (Addo et al. 2022; Li et al. 2018; Wijsman et al. 2019; Williams 2017), and the recent isolation of microplastics in various human organs and excreta (Huang et al. 2022; Pironti et al. 2022; Ragusa et al. 2021, 2022; Yan et al. 2021; Zhang et al. 2021), it is important that studies on potential exposure pathways are intensified to broaden global understanding on the extent of the problem and as well mitigating the problem. A recent review by Wootton et al. (2022), however, showed there are only 49 studies that investigated the presence of microplastics in oysters globally. Further analysis of a subset of 29 out of the 49 studies showed that microplastics were present in all oysters sampled globally, yielding a global average of 1.41 ± 0.33 MPs/gw (Wootton et al. 2022). A striking outcome of this review, however, is the paucity of data on studies on microplastics in oysters from all continents except Asia where most such studies have focused. Mostly, these studies focused on cultured oysters and not wild-caught ones. The outcome of this review amplifies the need to intensify microplastic research on oysters in data-deprived regions such as Africa and South America. While there are quite a few studies on microplastics in coastal environments from Africa (Adika et al. 2020; Ana Isabel Catarino et al. 2023; Chico-Ortiz et al. 2020; Mahu et al. 2023; Nchimbi et al. 2022; Pereao et al. 2020; Preston-Whyte et al. 2021; Sparks 2020; Tata et al. 2020), only a few of such studies report microplastics in oysters (Addo et al. 2022; Awuor et al. 2020; Onyango 2020). Our study reports the occurrence and distribution of microplastics in the Mangrove oyster, *Crassostrea tulipa* sampled from The Gambia, Sierra Leone, Ghana, Benin, and Nigeria. For each country, we considered the abundance, shapes/types, and sizes of microplastics as well as the polymer compositions of the microplastics.

## Material and methods

### Study sites

**Fig. 1:**
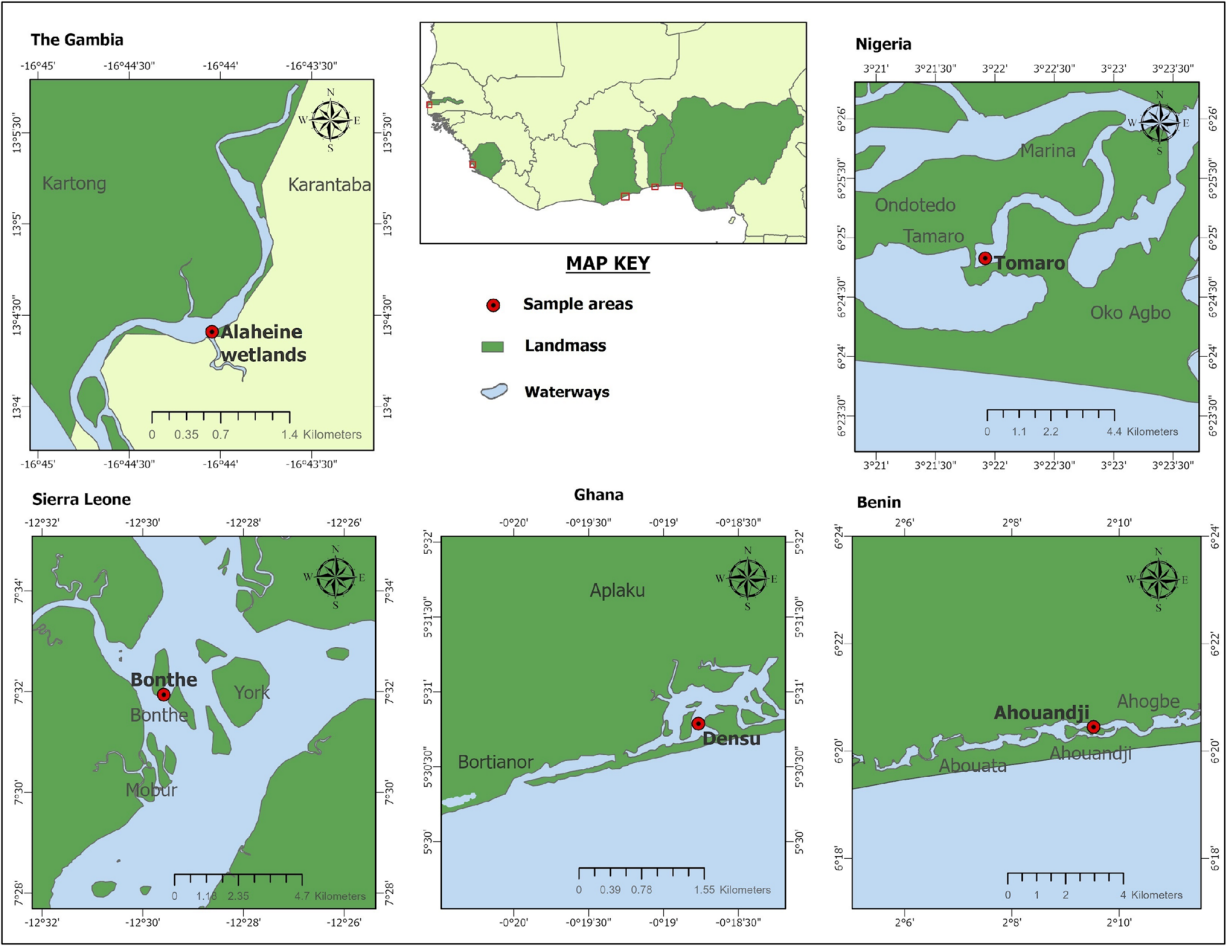
Map of *Crassostrea tulipa* sampling areas across West Africa

The study was carried out in the Tanbi/Alaheine Wetlands in The Gambia, the Bonthe/Sherbro estuary in Sierra Leone, the Densu estuary in Ghana, the Ouidah lagoon in Benin, and the Lagos lagoon in Nigeria (Fig. 1). Although mangrove oysters are typically found attaching to the roots of the red mangrove, oysters from Sierra Leone, and Ghana was sampled from shell substrates in the bottom of the estuaries while those from The Gambia, Benin, and Lagos were sampled from the roots of mangroves.

### Sample collection and preparation

Live oysters were collected from coastal waters of the Gambia, Sierra Leone, Ghana, Benin, and Nigeria between December 2020 and May 2021. Fifty individual oysters of different sizes were collected in different locations from each estuary. The oyster samples were wrapped in aluminum foil and transported on ice at 4 °C to the biogeochemistry laboratory at the Department of Marine and Fisheries Sciences at the University of Ghana and kept at − 20 °C until processing. Before analysis, samples were allowed to thaw at room temperature, and their morphometric characteristics (i.e., weight and total lengths) were recorded. The whole muscle of each oyster was put in a clean glass beaker and digested in 10% potassium hydroxide (KOH) at 40 °C for 72 h (Hara et al. 2020). The digested samples were filtered through a 1.2-μm glass membrane filter (CHMLAB–GF3). The filters were placed into clean glass Petri dishes, covered, and dried in an oven at 40 °C for 30 min, and later examined for microplastics. Thawing, measuring of morphometric characteristics, and digestion were carried out in a laminar flow hood.

### Isolation, identification, and characterization of microplastics

The dried filters were examined for microplastics under bright film using a stereomicroscope (Motic SMZ-171) with a digital camera (Moticam A-16) mount. The filter papers were examined under 40 × magnification in a zig-zag pattern to avoid overestimation of the count (Park & Park 2021). Microplastics were enumerated, and photographed, and their sizes and shapes were determined as fibers (long and elongated), fragments (irregular and angular pieces mainly from the degradation of large plastic materials), and films (clear and transparent) (Bessa et al. 2019). The sizes of microplastics were grouped as 51–100 μm, 101–500 μm, 501–1000 μm, 1001–5000 μm, and > 5000 μm. A Perkin Elmer spotlight 200i FTIR microscope system connected to a temperature-stabilized deuterated triglycine sulfate detector was used in the analysis of polymers in the isolated microplastics. FTIR differentiates various polymer types based on their unique chemical bonds and functional groups. This is crucial for distinguishing between different types of plastics, such as polyethylene (PE), polypropylene (PP), polystyrene (PS), and polyethylene terephthalate (PET), among others. Sample spectra were collected in transmission mode in 128 scans (minimum), with a spectral resolution of 4 cm^−1^, in a wavenumber range of 4000–400 cm^−1^. Scanning of MPs was preceded by measurements of background spectra to eliminate interference and reference polymers were used to validate the analysis. The OMNIC Spectra Software (Thermo Scientific) was used to identify microplastic polymers in each spectrum. Reference spectra ranging from 70 to 95% were used to match polymers. Data visualization was done in ORIGINLAB Professional. The FTIR polymer analysis was carried out on 10% of the total number of microplastics counted in each estuary. Although increasing the number of polymer composition analyses would have been ideal for the representation and liability of the results obtained (reference to polymer types), we sampled 10% of the total number of microplastics in line with the guidelines of the Marine Strategy Framework Directive (MSFD) technical group on marine litter (Galgain et al. 2013).

### Quality control and assurance

As a precaution to reduce the risk of cross-contamination, only cotton laboratory coats and nitrile gloves were used throughout the study. We deployed the use of metal trays and instruments for shucking the oysters to avoid procedural contamination from plastic materials. Laboratory glassware was decontaminated with 10% nitric acid for 24 h, rinsed several times with filtered distilled water (0.45 μm), and covered immediately with aluminum foil to prevent air particle accumulation. Air controls were used at every stage of the procedure. Procedural blanks were performed on potassium hydroxide solutions to check potential contamination from the extraction solution. Commercial polymers of different sizes were purchased for matrix spike to evaluate the efficiency of the analytical method. Oyster tissues were spiked with 10 particles each of polypropylene (PP), polyethylene (PE), and expanded polystyrene (EPS), and then tested using the method described in Cho et al. (2019). The percentage recovery was 99% for polyethylene, 100% for polypropylene, and 99% for polystyrene.

### Data analysis

GraphPad Prism version 9.5 was used to analyze and visualize microplastic abundance, size, and averages. Non-parametric statistical analysis was performed based on the outcome of the descriptive normality test. Microplastic abundances were analyzed using a Kruskal–Wallis test for analysis of variance, followed by Dunn’s test for multiple comparisons. The level of significance was set at *α* = 0.05.

### Results and discussion

#### Microplastic abundance and distribution

**Fig. 2:**
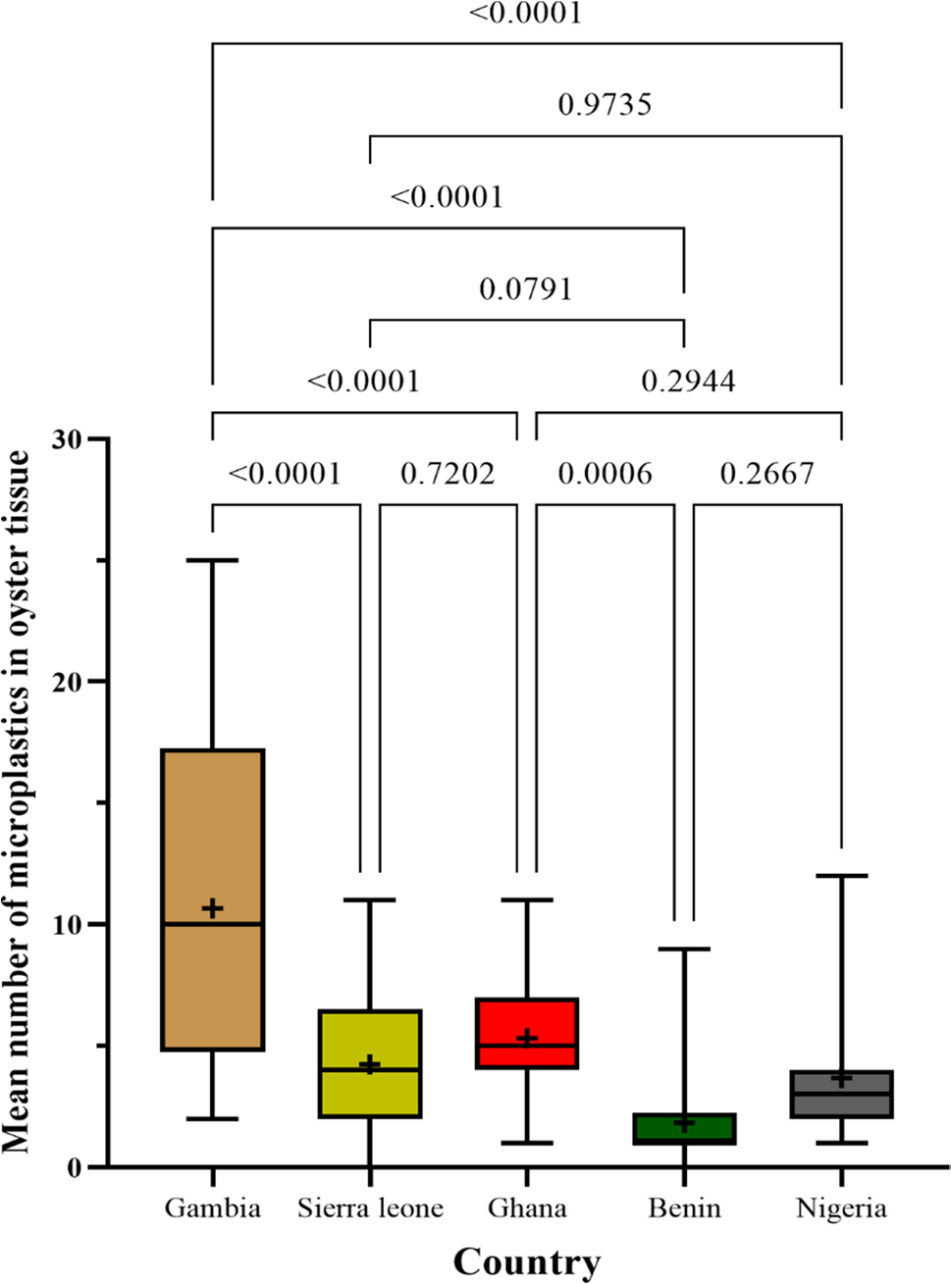
Average numbers of microplastics isolated in tissues of *Crassostrea tulipa* from estuaries in The Gambia, Sierra Leone, Ghana, Benin, and Nigeria. A pairwise comparison shows a significant difference between countries, i.e., *p*-value > 0.05

Data reported are from the total digestion of whole muscle. Microplastics were detected in 180 (72%) of the 250 oysters studied. A total of 780 microplastics were isolated in the whole tissues of the oysters. Gambia recorded the highest microplastic abundance (315) followed by Ghana (177), Sierra Leone (120), Nigeria (114), and finally Benin (54). The average number of microplastics per oyster varied significantly across the five countries (*p* < 0.05; Fig. 2). Just like the trend observed with abundance, the Tanbi wetlands in the Gambia recorded the highest average per oyster (10.50 ± 6.69) followed by the Densu estuary in Ghana (5.90 ± 2.40), Sherbro estuary in Sierra Leone (4.14 ± 2.92), Lagos lagoon in Nigeria (3.68 ± 2.45), and the Ouidah lagoon in Benin (1.80 ± 1.90) (Table 1). Seventy-two percent of the total population had ingested at least one microplastic. The percentage occurrence was highest for Gambia (84) and the lowest was observed for Benin (60%) (Table 2).

**Table 1:** Location, shell morphometry, and average numbers of microplastics in *Crassostrea tulipa* across the five countries

Country	Estuary/lagoon	Shell length (cm)	Shell width (cm)	Tissue weight (g)	Average MP per individual
Gambia	Alaheine	5.54 ± 1.09	3.78 ± 0.85	1.96 ± 0.66	10.50 ± 6.69
Ghana	Densu	5.13 ± 1.15	1.25 ± 0.53	2.88 ± 1.12	5.90 ± 2.40
Benin	Ouidah	5.58 ± 1.04	1.62 ± 0.50	1.73 ± 0.94	1.80 ± 1.90
Nigeria	Lagos	4.86 ± 1.41	1.24 ± 0.58	1.79 ± 1.01	3.68 2.45
Sierra Leone	Sherbro	6.28 ± 2.07	1.25 ± 0.54	3.18 ± 1.72	4.14 ± 2.92

**Table 2:** Percentage occurrence of microplastics in oysters across the five countries

Country	Number of individuals examined	Number of positive individuals	Number of negative individuals	% Occurrence
Ghana	50	40	10	80
Nigeria	50	33	17	66
Benin	50	30	20	60
Gambia	50	42	8	84
Serre Leone	50	35	15	70
Total	250	180	70	72

Dunn’s multiple comparisons tests yielded a significant mean rank difference between the Gambia and Sierra Leone (*p* = 0.000), the Gambia and Benin (*p* < 0.0001), Sierra Leone and Benin (*p* = 0.0124), the Gambia and Nigeria (*p* < 0.0001), and Sierra Leone and Ghana and Benin (*p* < 0.0001). On the other hand, the mean rank differences were not significant between the Gambia and Ghana (*p* = 0.642), Sierra Leone and Ghana (*p* = 0.320), Sierra Leone and Nigeria (*p* > 0.999), Ghana and Nigeria (*p* = 0.0885), and Benin and Nigeria (*p* = 0.059). The variabilities in average microplastic numbers in oysters observed in this study are likely to result from a variety of factors including the size/age and width of the shell, attachment substrate, and the state of their environments. Wu et al. (2022) observed a direct relationship between the intake of microplastic by clams and their shell size with microplastic counts increasing by 1.01 times for every 1-mm increase in shell width. Generally, the oysters studied showed considerable variabilities in their shell lengths and widths with only the Gambian oysters having comparatively wider shell widths than those from the other countries (Table 1). Size plays a role in the filtration efficiency and feeding rates of bivalves with larger bivalves having a competitive advantage over smaller bivalves (Webb et al. 2013). In addition to the size, the attachment substrate of the oysters could also account for the variability of microplastics observed in the oysters across the countries. Although *C. tulipa* can be found on diverse substrates, the most utilized attachment substrate is the root of red mangroves which abounds in their habitats (Mahu et al. 2022).

The oysters from Sierra Leone, and Ghana, however, were collected from shell substrates submerged in water in the Sherbro, and Densu estuaries respectively while those from The Gambia, Lagos, and Benin were collected from the roots of red mangroves in intertidal areas of the Lagos and Ouidah Lagoon respectively. Unlike the shell substrates which are always submerged fully in the water, the roots of the mangroves get exposed periodically during the low tides, thereby reducing the exposure time and duration of the oysters to microplastics as well as the filtration frequency from the water. These periodic exposures imply that the oysters attached to the mangrove roots only get exposed to microplastics in water when the mangrove roots get submerged in the water. In the case of The Gambian oysters, intense fishing activities could be a possible reason for the high numbers of microfibers counted as the Tanbi wetlands host one of the major fish landing sites in Gambia. In addition, the time of sampling of the oysters could have contributed to the variability in the number of microplastics counted. While the Ghana, Benin, and Nigeria samples were collected during the dry season, samples from Gambia and Sierra Leone were collected in the wet season where large numbers of land-based plastics are washed into estuaries. Finally, the microplastic load of the surrounding water in which the oysters live may contribute to the variabilities observed as well as the age of the oysters. Older oysters have higher exposure duration to contaminants compared to younger and juvenile ones (Bernal 2020). The microplastic numbers reported in oysters from all five (5) countries in this study are similar and comparable to those obtained in other places around the world (Blosser 2022; Cole & Galloway 2015; Li et al. 2018; Liao et al. 2021; Patterson et al. 2019; Teng et al. 2019).

### Microplastic shapes

**Fig. 3:**
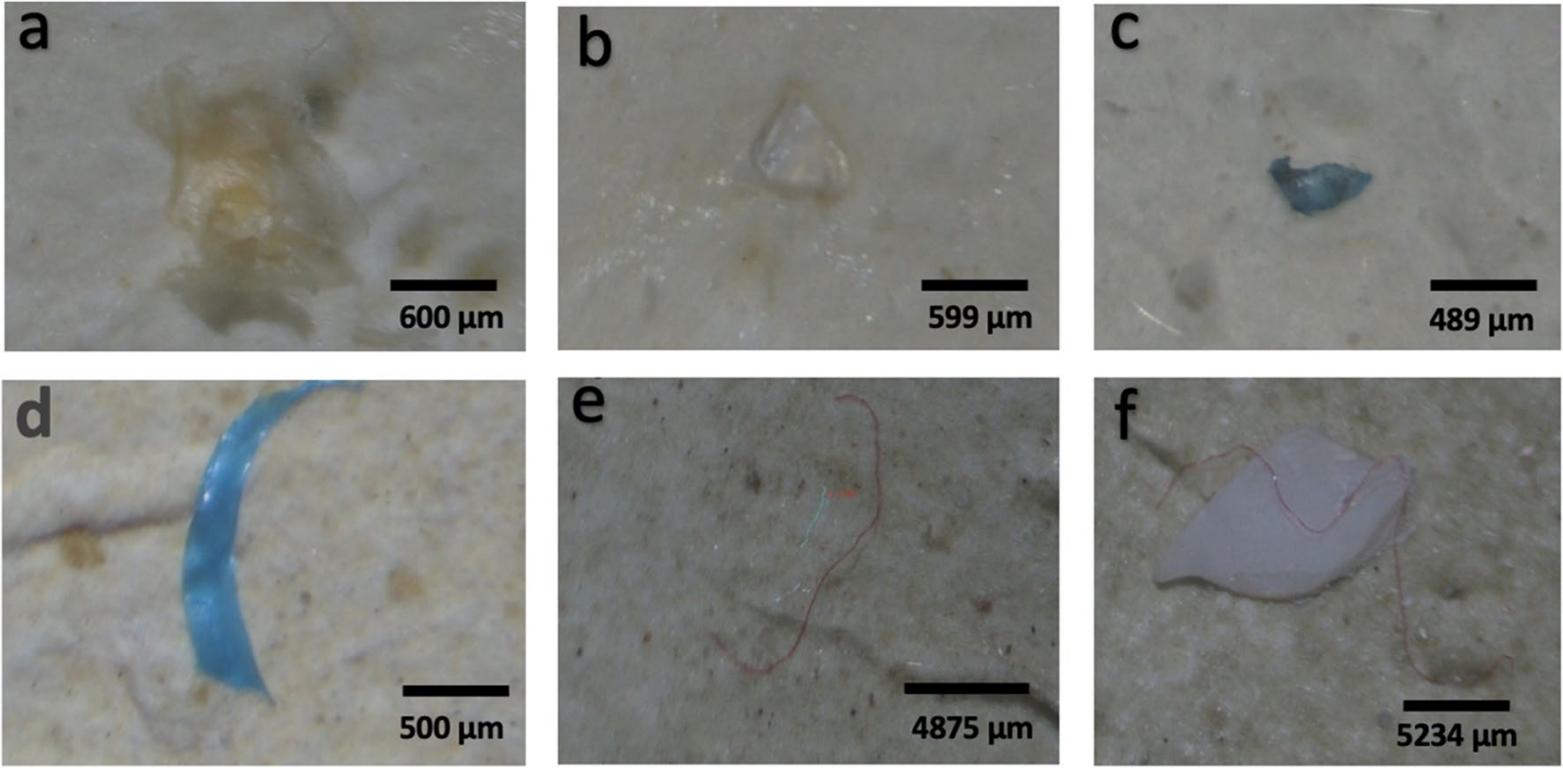
Examples of microplastics identified in *C. tulipa* muscles from West Africa (**a** film plastics; **b**–**d** plastic fragments; **e**–**f** microfibers)

**Fig. 4:**
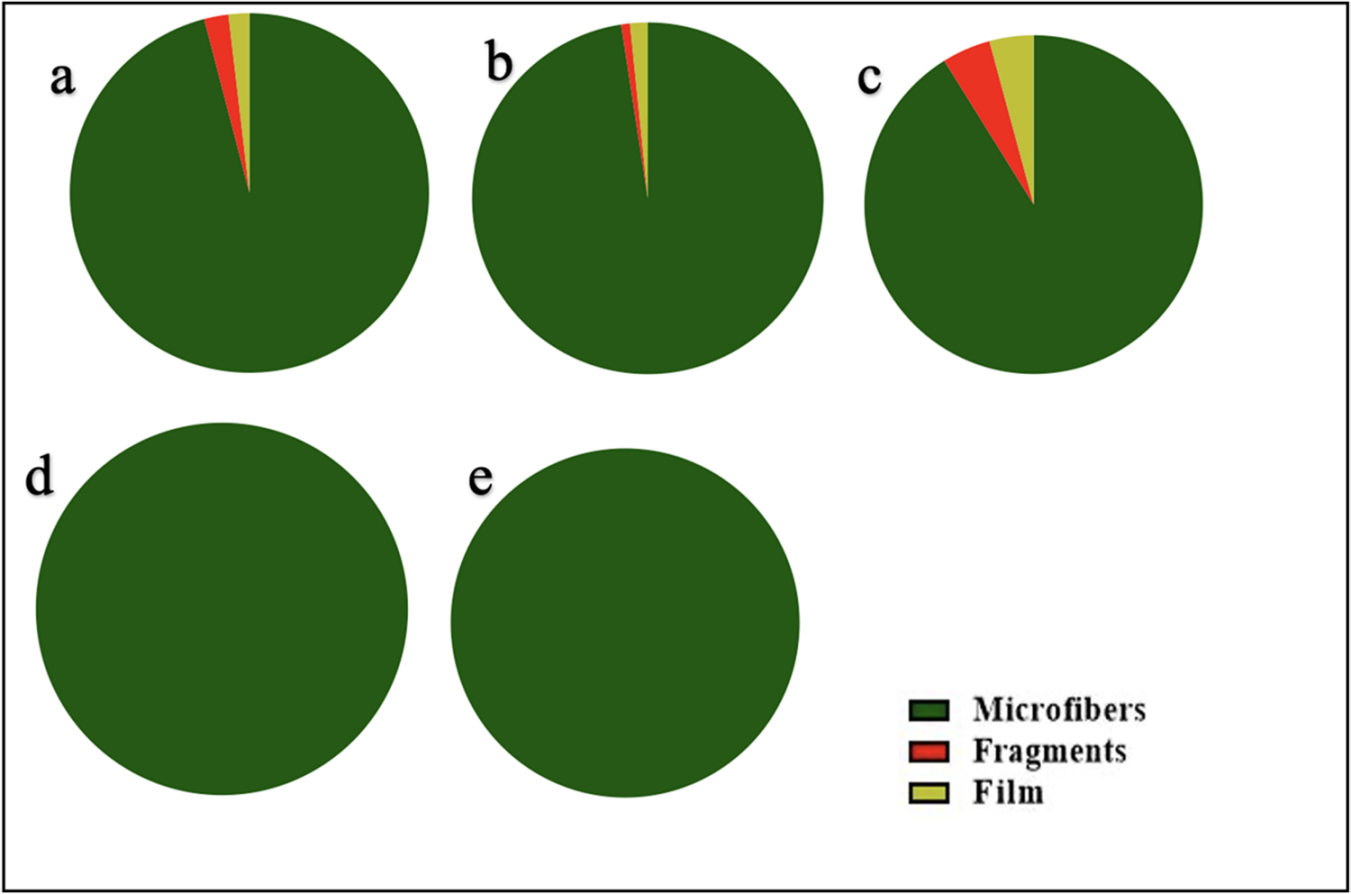
Abundance and distribution of microplastic types/shapes in *Crassostrea tulipa* from Gambia (**a**), Sierra Leone (**b**), Ghana (**c**), Benin (**d**), and Nigeria (**e**)

The toxicity of microplastics to marine organisms depends on their shape, making shape analysis an important component of microplastic research. We observed three (3) shapes/types of microplastic namely, microfibers, fragments, and films (Fig. 3). Of these, microfibers dominated all the counts in the oysters from all five (5) countries (Fig. 4). The oysters from Nigeria and Benin had a 100% microfiber composition. This was followed by the Sierra Leone oysters with a 98% microfiber composition, The Gambia with a 96% microfiber composition, and Ghana with a 91% microfiber composition. Fragments and films were present in very small numbers (5% and 4% respectively) for Ghana (2% and 2% respectively) for the Gambia, and (2% and 1% respectively) for Sierra Leone. The oysters from the Densu estuary in Ghana had more fragments and films than those from the Tanbi wetlands and Sherbro estuary. In agreement with the findings of this study, several studies have reported large numbers of microfibers in oyster tissue compared to other microplastic types (Addo et al. 2022; Lozano-Hernández et al. 2021; Teng et al. 2019; Vieira et al. 2021; Waite et al. 2018). The high uptake of microfibers by the oysters could be explained within the context of the prevalence of microfibers in the marine environment compared with other microplastics or anatomical constraints of oysters such gill, labial palps, and mouth which are found to reduce the likelihood of ingestion of other shapes of microplastics such as microspheres.

Approximately 2,000,000 tonnes of microfibers are released into the ocean every year from various sources, of which 700,000 comprise fleeces released per garment through domestic laundry activities (Mishra et al. 2019). The rate of disintegration of fibers from garments into the environment increases with worn-out or mishandled clothing as well as upon exposure to environmental conditions (Mishra et al. 2021). Africa ranks as the number one (1) importer of the lowest grade of second-hand clothing worldwide (Manieson & Ferrero-Regis 2022). Most of these second-hand clothing come in very poor and overly worn-out conditions with over 40% of such items in West Africa ending up in landfills. Coupled with incompetent waste management measures, these worn-out fabrics find their way into the coastal environment where they shred into several tiny microfibers under varying ocean conditions. Once these microplastics get into the estuaries and lagoons, oysters which have the potential to filter as much as 50 gallons of water in a seawater day while feeding (Kecinski et al. 2015), ingest the free-floating microfibers in very large numbers. Additionally, the high retention of microfibers by oysters when consumed compared to other microplastic types could also account for the dominance of microfibers observed in our study. Ward et al. (2019) reported high egestion of spherical microplastic compared to fibers in the *C. gasar.*

Microfiber ingestion by marine organisms has negative implications for their overall physiological health and functioning. Although specific studies looking at the effect of microfiber ingestion on oysters are limited, studies on other similar filter-feeding organisms such as crabs have reported various detrimental effects. For example, Watts et al. (2015) reported a statistically significant switch from positive to negative growth and reduction in food consumption by *C. maenas* exposed to polypropylene microfibers for 4 weeks. Woods et al. (2018) reported a significant reduction in filtration rates by the blue mussel (*Mytilus edulis*) exposed to microfibers in a laboratory experiment. Mussels that were not exposed to microfiber had an average filtration rate of 50.1 mL min^−1^, while those that were exposed had an average filtration rate of 23.9 mL min^−1^. Similar studies observed a triggered production of pseudofeces and the complete closure of feeding valves by mussels that were exposed to very high concentrations of microfibers (Wegner et al. 2012). Reduction in filtration rate and efficiency in bivalves presents them with physiological challenges such as growth and reproduction inefficiencies. In addition to organismal and ecosystem level impacts, oysters are popular seafood consumed whole, without gut removal, hence, can be a direct route of human exposure to microplastics. Ding et al. (2022) estimated the global mean value of microplastic exposure to humans via mollusk consumption to be 751 microplastics/per capita/per year, echoing the importance of mollusks including oysters as an important exposure route of microplastics to humans under non-depurated conditions.

### Microplastic sizes

**Fig. 5:**
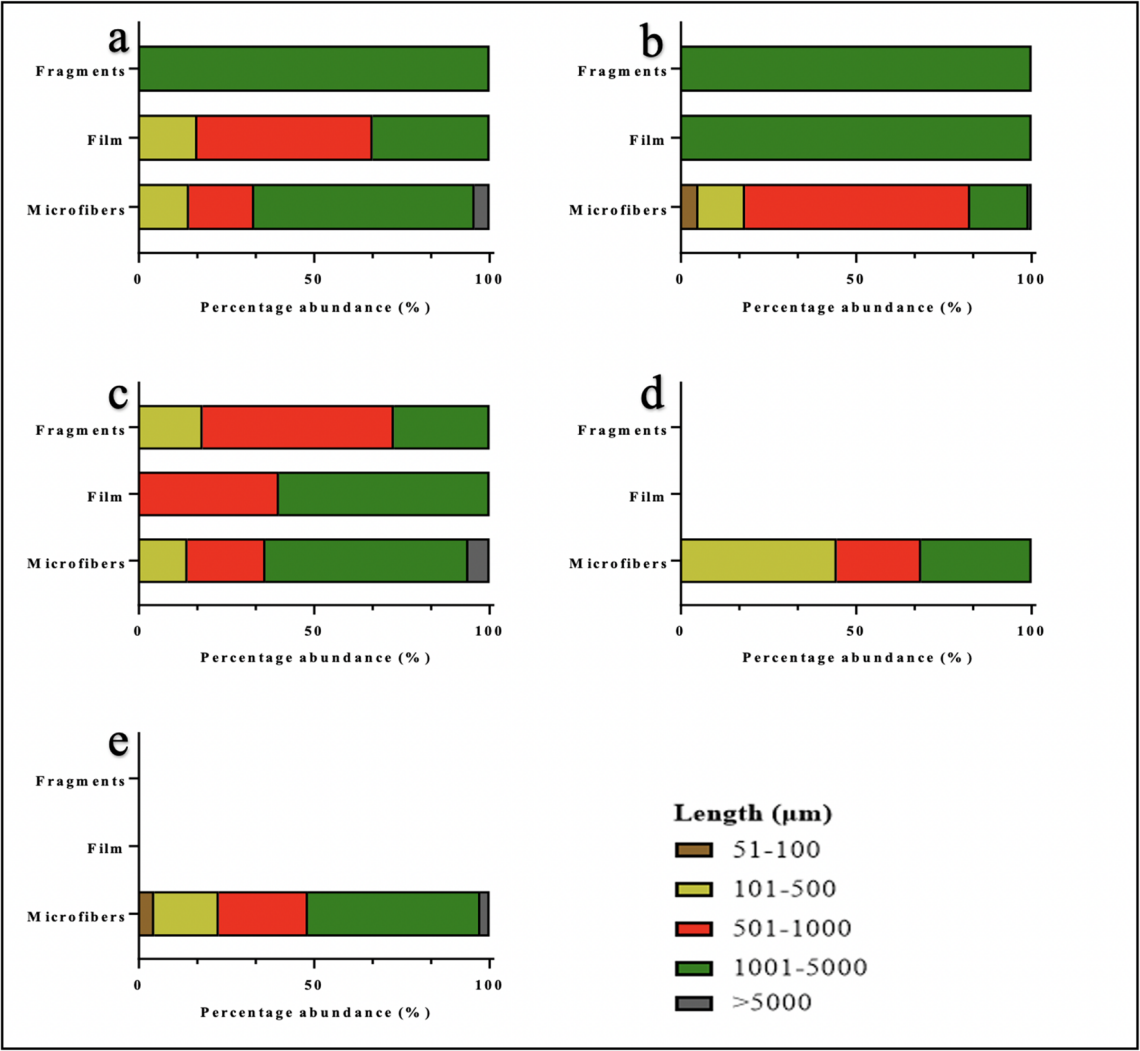
Sizes of microplastics ingested by *Crassostrea tulipa* from The Gambia (**a**), Sierra Leone (**b**), Ghana (**c**), Benin (**d**), and Nigeria (**e**)

We observed six classes of size for the different shapes of microplastics analyzed across the five countries (Fig. 5). Microplastic between the 1001 and 5000 μm size class was the dominant and prevalent size class observed across all three (3) shapes identified. This was followed by 501–1000 μm, 101–500 μm, and 51–100 μm. A very small percentage of microfibers in oysters from Sierra Leone, Ghana, and Nigeria had size classes greater than 5000 μm, making them fall outside of the size definition of microplastics. Except for microfibers from Sierra Leone and Nigeria, microplastic within the size class of 51–100 μm was completely absent in all shapes isolated in the oysters from the Gambia, Ghana, and Benin. Several studies have established a direct relationship between microplastic size and biological effects (Jeong et al. 2016, 2017; Lei et al. 2018; Lu et al. 2016). So far, the greatest effects have been observed for the smallest diameter particles due to the prolongation of the retention times and their higher bioavailability (Prokić et al. 2019). While most studies demonstrating the toxicity of microplastics to marine organisms have focused on the smallest size classes (5 to 25 μm) due to their ability to translocate guts into various organs, it is important to note that the size classes observed in this study, particularly, those between 51 and 100 μm are quite relevant within the context of toxicity to humans. The reason is that oysters are consumed as a whole, hence, these microplastics of varying sizes may enter the human body directly where they are likely to interact with various organs to cause toxic effects. There is evidence suggesting that the primary toxins in microplastics such as dyes and plasticizers are toxic, carcinogenic, and mutagenic (Gasperi et al. 2018). In the environment, microplastics interact with a wide array of pollutants such as persistent organic pollutants and heavy metals which get adsorbed onto their surfaces (Gao et al. 2022; Song et al. 2022; Yang et al. 2022a, b). Once consumed, these toxic pollutants may become bioavailable to humans, producing varying levels of toxic effects.

### Polymer types

**Fig. 6:**
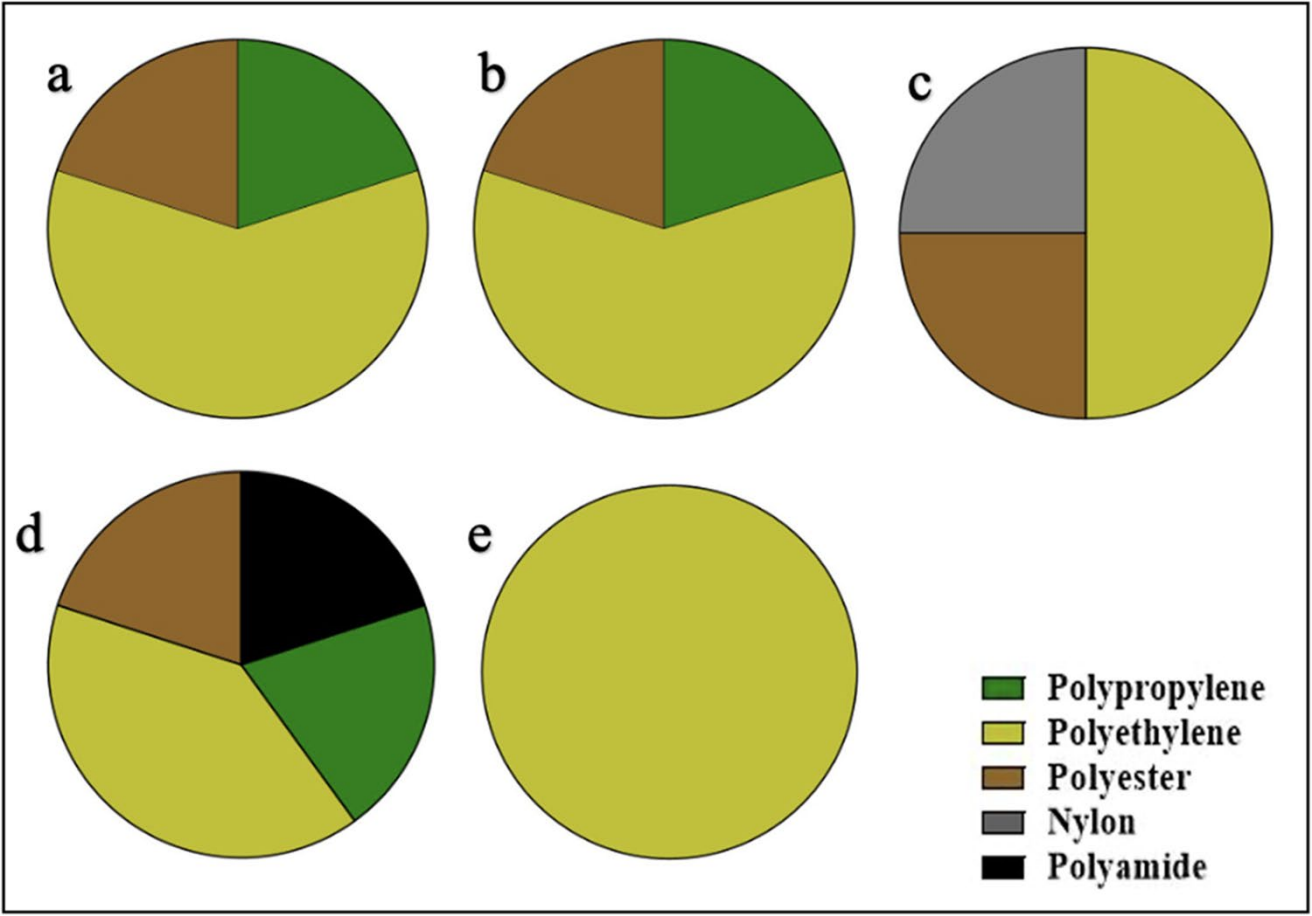
Polymer types identified in microplastics ingested by *Crassostrea tulipa* from The Gambia (**a**), Sierra Leone (**b**), Ghana (**c**), Benin (**d**), and Nigeria (**e**)

**Fig. 7:**
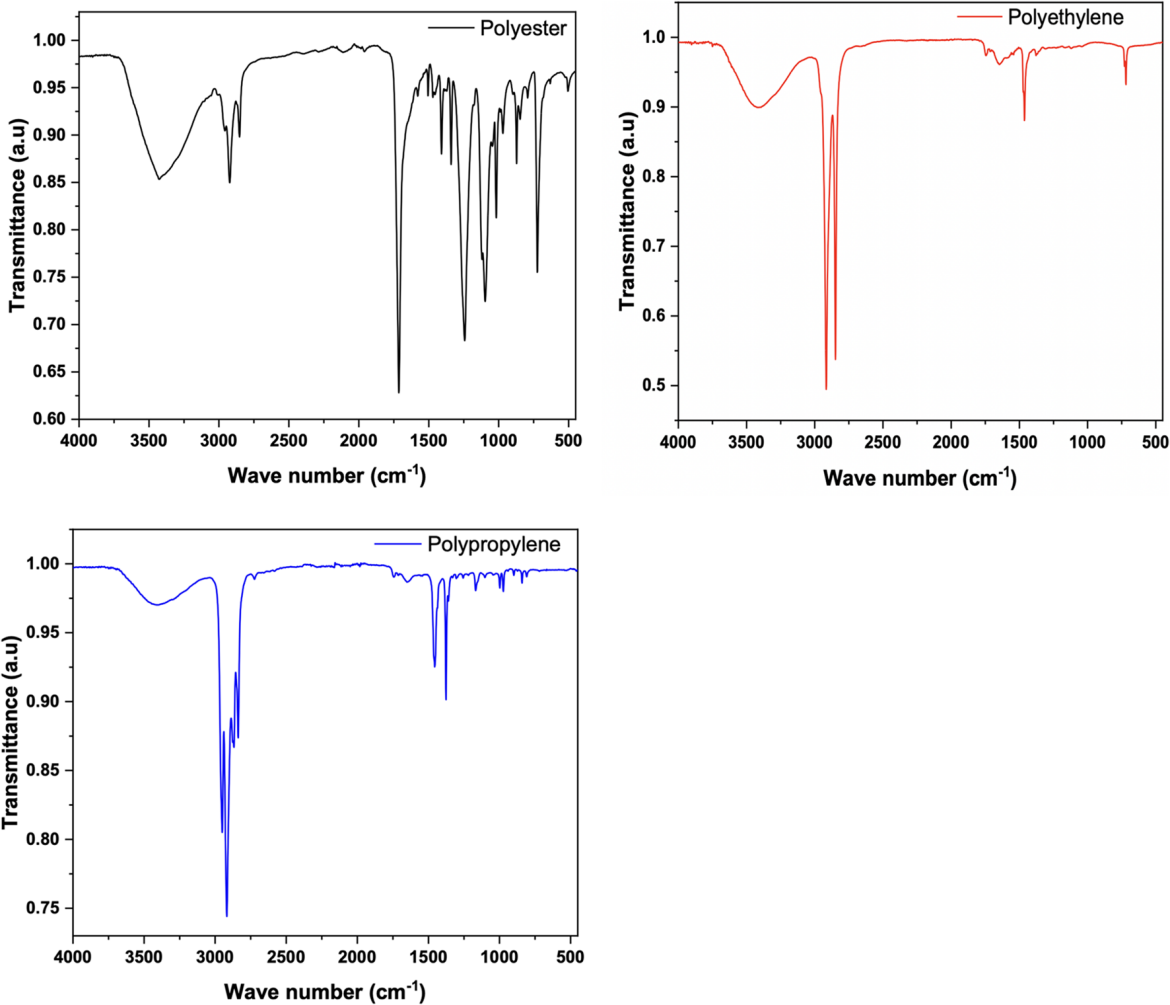
Examples of spectral peaks obtained for polyester, polyethylene, and polypropylene

Five polymer types were identified from the polymer analysis (Fig. 6). Spectral peaks of a selection of polymers identified are presented in Fig. 7. These were nylon, polyester, polyethylene, polypropylene, and polyamide. Of these, polyethylene occurred in the microplastics isolated in oysters from all five countries, whereas polyester was identified in oysters from all countries except Nigeria. Nylon was only present in the oysters from Ghana. Polyamides only occurred in the oysters from Benin. Polypropylene was missing in the Ghana and Nigeria samples. The highest polymer diversity was observed in the Benin oysters with polyethylene dominating polymer composition in all countries.

The high polymer diversity in oysters observed across and within the countries suggests varying sources of microplastic, especially microfiber input into the environments of the oysters. The polymers identified in this study are commonly used in fishing nets, synthetic fabrics, toys, disposable containers, plastic films, plastic bags, and bottles, hence not surprising that they were prevalent in oyster tissues. The polymers observed in this study are comparable with those observed in other studies (Adika et al. 2020; Mahu et al. 2023; Makhdoumi et al. 2021). The prevalence of high-density polyethylene polymers in fish from coastal environments in West Africa has been reported in other studies (Mahu et al. 2023; Pappoe et al. 2022). Surveys on large plastic items on beaches in West Africa reveal the dominance of single-use plastic items such as polyethylene water sachets, polyethylene carrier bags, PET bottles, and cups (Ana I Catarino et al. 2023; Fadare et al. 2022; Nukpezah et al. 2022). It is therefore not surprising that polyethylene dominates microplastic types consumed by oysters. The occurrence of polyester-sourced microplastics in seafood has been reported for the first time in this study for West Africa. Polyesters and polyamides are among the most important synthetic fibers, accounting for about 60% of the total world fiber production, and are used widely in the production of clothing (Šaravanja et al. 2022). Largely, polyester fibers are released from fabrics during laundry and discharged into the environment (Dalla Fontana et al. 2020; Hernandez et al. 2017; Šaravanja et al. 2022). In addition to laundry, used clothing disposed into the coastal environment may shed polyester fibers as they degrade (Liu et al. 2021). As noted in preceding sections, West Africa has become a global trade hub for second-hand clothes, most of which come in very deteriorated conditions that get disposed into the environment (Manieson & Ferrero-Regis 2022). In Ghana, for instance, large patches of disposed second-hand clothing debris are found either buried or lying on the beaches (Manieson & Ferrero-Regis 2022; Van Boeckholtz 2020) where they break down and shed materials including fibers into the environment over time. Laboratory and field studies are currently ongoing to understand the extent to which these second-hand clothing shed fibers into Ghana’s coastal environment. While the study provides important data on polymer composition and distribution in shellfish from the coastal waters of West Africa, we acknowledge some challenges in the isolation of microplastic particles for polymer identification for the model of FTIR used. Only particles greater than 500 μm were subjected to FTIR analysis, therefore, an advanced technique would have been beneficial to include smaller particles.

## Conclusion

Microplastic pollution of coastal and marine ecosystems is a threat to marine organisms, ecosystem functioning, and human health. From the disruption of physiological processes in ecosystems to long-term health impacts in humans, there is a strong need for data to understand the extent of the problem, propose solutions, and sensitize the public on seafood safety. This study, therefore, aimed to investigate the extent of microplastic contamination in the muscles of the West African Mangrove Oyster, *Crassostrea tulipa*, focusing on microplastic abundance, shape, size, and polymer in samples obtained from five countries in West Africa.

The total number of microplastics varied significantly among the five countries with the oysters from the Gambia recording the highest numbers of microplastics whereas those from Benin recorded the least. Oysters sampled from the shell substrates in the bottom waters recorded higher loads of microplastics compared to those sampled from mangrove roots, except in the case of the Gambia. The oysters from this study present some of the heavy microplastic loads in comparison with globally reported data in oysters.

Throughout the five countries, microfibers of varying sizes dominated the counts. Of the four classes of sizes identified, microfibers within the size range of 1001–5000 μm dominated the counts while those in the size class of 51–100 μm were the least encountered and occurred in only the Sierra Leone and Nigerian oysters. The ingestion of both large and smaller microplastics by oysters presents feeding challenges as well as toxic implications for them. Consumption of oysters by humans may expose them to these varied size classes and associated contaminants absorbed to the surfaces of the microplastics with respective health implications.

Five polymer groups were identified for the microplastics isolated across the five countries with polyethylene being the dominant polymer. The higher diversity of polymers reported in the study suggests a diverse source of microplastics in the habitats of the oysters. These sources may include single-use PET plastics and clothing including second-hand ones.

Generally, the microplastic loads observed in the oysters were higher than those reported in most studies globally and this suggests one thing, that the coastal environments in West Africa are among the ones with heavy loads of plastics. This observation is reported widely in the literature as well as visually. It is important that in the face of complex environmental change, where marine organisms are constantly under stress from climate variabilities, additional stressors such as pollution from plastics are critically minimized to build resilience within such ecosystems. We recommend management measures that that target potential sources of microfibers such as discarded or abandoned fishing nets and used clothing which contribute largely to fiber pollution.

## Data Availability

The authors agree to share the research data upon request.
